# Pathogenic Gene Prediction Algorithm Based on Heterogeneous Information Fusion

**DOI:** 10.3389/fgene.2020.00005

**Published:** 2020-02-04

**Authors:** Chunyu Wang, Jie Zhang, Xueping Wang, Ke Han, Maozu Guo

**Affiliations:** ^1^School of Computer Science and Technology, Harbin Institute of Technology, Harbin, China; ^2^School of Computer and Information Engineering, Harbin University of Commerce, Harbin, China; ^3^School of Electrical and Information Engineering, Beijing University of Civil Engineering and Architecture, Beijing, China; ^4^Beijing Key Laboratory of Intelligent Processing for Building Big Data, Beijing University of Civil Engineering and Architecture, Beijing, China

**Keywords:** pathogenic gene prediction, induction matrix completion, compact feature learning, PU-Learning, mean percentile ranking

## Abstract

Complex diseases seriously affect people's physical and mental health. The discovery of disease-causing genes has become a target of research. With the emergence of bioinformatics and the rapid development of biotechnology, to overcome the inherent difficulties of the long experimental period and high cost of traditional biomedical methods, researchers have proposed many gene prioritization algorithms that use a large amount of biological data to mine pathogenic genes. However, because the currently known gene–disease association matrix is still very sparse and lacks evidence that genes and diseases are unrelated, there are limits to the predictive performance of gene prioritization algorithms. Based on the hypothesis that functionally related gene mutations may lead to similar disease phenotypes, this paper proposes a PU induction matrix completion algorithm based on heterogeneous information fusion (PUIMCHIF) to predict candidate genes involved in the pathogenicity of human diseases. On the one hand, PUIMCHIF uses different compact feature learning methods to extract features of genes and diseases from multiple data sources, making up for the lack of sparse data. On the other hand, based on the prior knowledge that most of the unknown gene–disease associations are unrelated, we use the PU-Learning strategy to treat the unknown unlabeled data as negative examples for biased learning. The experimental results of the PUIMCHIF algorithm regarding the three indexes of precision, recall, and mean percentile ranking (MPR) were significantly better than those of other algorithms. In the top 100 global prediction analysis of multiple genes and multiple diseases, the probability of recovering true gene associations using PUIMCHIF reached 50% and the MPR value was 10.94%. The PUIMCHIF algorithm has higher priority than those from other methods, such as IMC and CATAPULT.

## Introduction

The discovery of disease-causing genes plays an important role in understanding the causes of diseases, clinically diagnosing diseases, and achieving early prevention and treatment (Cheng et al., 2016; Zeng et al., 2017; Cheng et al., 2019). It is also an important goal of human genome research, with great scientific and social significance. Prioritization of potentially pathogenic genes is an important step in the discovery of disease-causing genes and obtaining an understanding of genetic diseases.

Early studies of gene–disease associations were based on clinical and biological experiments, which are expensive and time-consuming. Owing to the inherent difficulties and delays in the study of human genetic diseases, there are very few known identified gene–disease links in public databases, such as the widely used Online Mendelian Inheritance in Man (OMIM) (Amberger et al., 2015) and Genetic Association Database (Becker et al., 2004). Because of the specificity of the study of disease-causing genes, we do not know the genes that are not related to a particular disease. We only know the few genes that have been proven to be related to it. Against this background, with the emergence of bioinformatics, researchers have begun to focus on and study genetic disease prioritization algorithms, and use computer technology to mine candidate pathogenic genes from massive data (Liu et al., 2020; Wang et al., 2018; Zeng et al., 2018; Zhang et al., 2019; Zeng et al., 2019; Pan et al., 2019). The selected genes are more likely to be related to diseases, and gene sorting algorithms with better predictive performance would be more helpful to conduct targeted biological experiments and understand pathogenic mechanisms.

Early gene sorting algorithms based on network similarity focused on local information in the gene–disease network, namely, nodes adjacent to gene or disease nodes; an example of these is the molecular triangulation method (Krauthammer et al., 2004). It has been found that the global topology of a network can improve the performance in predicting disease-causing genes (Pan et al., 2019; Chen et al., 2019). Kohler et al. (Kohler et al., 2008) used the random walk (RWR) algorithm to analyze candidate disease-causing genes, which further improved the predictive performance.

Complex biological systems cannot always meet the needs of analysis with single network data (Chen et al., 2019). The continuous growth of biological data, such as high-throughput sequencing, also brings opportunities to study new predictive methods. The more commonly used databases include the gene expression database GEO (Barrett et al., 2007), the cancer gene information TCGA database (Cancer Genome Atlas Research et al., 2013), the protein interaction network database STRING (Szklarczyk et al., 2017), the Gene Ontology (GO) database (Ashburner et al., 2000), and Disease Ontology (DO) (Schriml et al., 2012). Recently, there has been increasing interest in studying gene sorting algorithms and starting to integrate a large amount of biological data and analyze heterogeneous networks (Gomez-Cabrero et al., 2014; Jiang, 2015; Zhang et al., 2019; Deng et al., 2019). In 2008, the CIPHER algorithm (Wu et al., 2008) was proposed by Wu et al., which combines protein interaction and disease-like networks but only considers local information in the network and lacks global topology. In 2010, Vanunu et al. (Vanunu et al., 2010) proposed the PRINCE algorithm, based on the idea of global network information and network dissemination. In the same year, Yongjin Li et al. (Li and Patra, 2010) proposed the restarted random walk algorithm (RWRH) that fused a gene similarity network, a disease phenotypic similarity network, and a large heterogeneous network composed of a disease phenotype–gene relationship network. In addition, Singh-Blom et al. (Singh-Blom et al., 2013) further improved the predictive performance in 2013 using the Katz method commonly used in the field of social networks for the task of predicting gene–disease relationships.

With the rapid development of machine learning and artificial intelligence in recent years, new algorithms based on machine learning have been applied to predict candidate pathogenic genes; they have shown good predictive performance (Zou et al., 2018; Peng et al., 2018; Liao et al., 2018; Zhang et al., 2018; Xiong et al., 2018; He et al., 2018; Cheng et al., 2018; Cheng et al., 2018; Zeng et al., 2019; Ding et al., 2019; Liu, 2019; Liu et al., 2019a; Zhu et al., 2019). In 2011, Mordelet et al. (Mordelet and Vert, 2011) considered the problem of genetic prediction as a supervised machine learning problem and proposed the ProDiGe method. Moreover, in 2013, Singh-Blom et al. (Kohler et al., 2008) proposed the supervised machine-learning method CATAPULT using a variety of data sources. Then, Natarajan et al. (Natarajan and Dhillon, 2014) applied the inductive matrix completion algorithm (IMC) in the recommendation system to predict pathogenic genes. This algorithm can not only predict existing genes and diseases but also predict new genes and diseases that have not previously been shown to be related. To compensate for the impact of a data sparseness and the PU problem, the PUIMCHIF algorithm is proposed in this paper. Specifically, on the basis of the original IMC algorithm, the main innovations and contributions of this paper can be summarized as follows: (1) owing to the sparsity of gene–disease association data, we used a variety of data sources to construct the characteristics of genes and diseases, and added a STRING data set for the compact feature learning of genes, which contained the physical relationships and other interactions that were not in the original data set. (2) For the gene–gene network and the disease–disease network (Li et al., 2019), we used the RWR method to obtain the diffusion state of each node in the network under a steady state in accordance with the network topology, used diffusion component analysis (DCA) to reduce the dimensions of the data, and finally obtained the network characteristics of genes or diseases. One advantage of this approach is the ability to analyze both HumanNet and STRING networks. (3) Self-encoders in machine learning can learn efficient representations of data for dimensionality reduction. Combined with the characteristics of biological data, the work described in this paper used denoising self-encoding to reduce the dimensionality of high-dimensional data features of genes and diseases. (4) Considering the sparse disease–gene association data and the prior knowledge that most unknown associations are negative cases, we adopted the PU-Learning strategy to treat unlabeled data as negative cases for biased learning, so as to replace the IMC method involving learning for only positive cases. (5) To verify the effectiveness of the PUIMCHIF method proposed in this paper, we used two commonly used evaluation indexes, Precision and Recall. On this basis, we added the MPR index of mean percentile ranking to further analyze the experimental results comprehensively.

## Introduction to Methods

**Figure 1:**
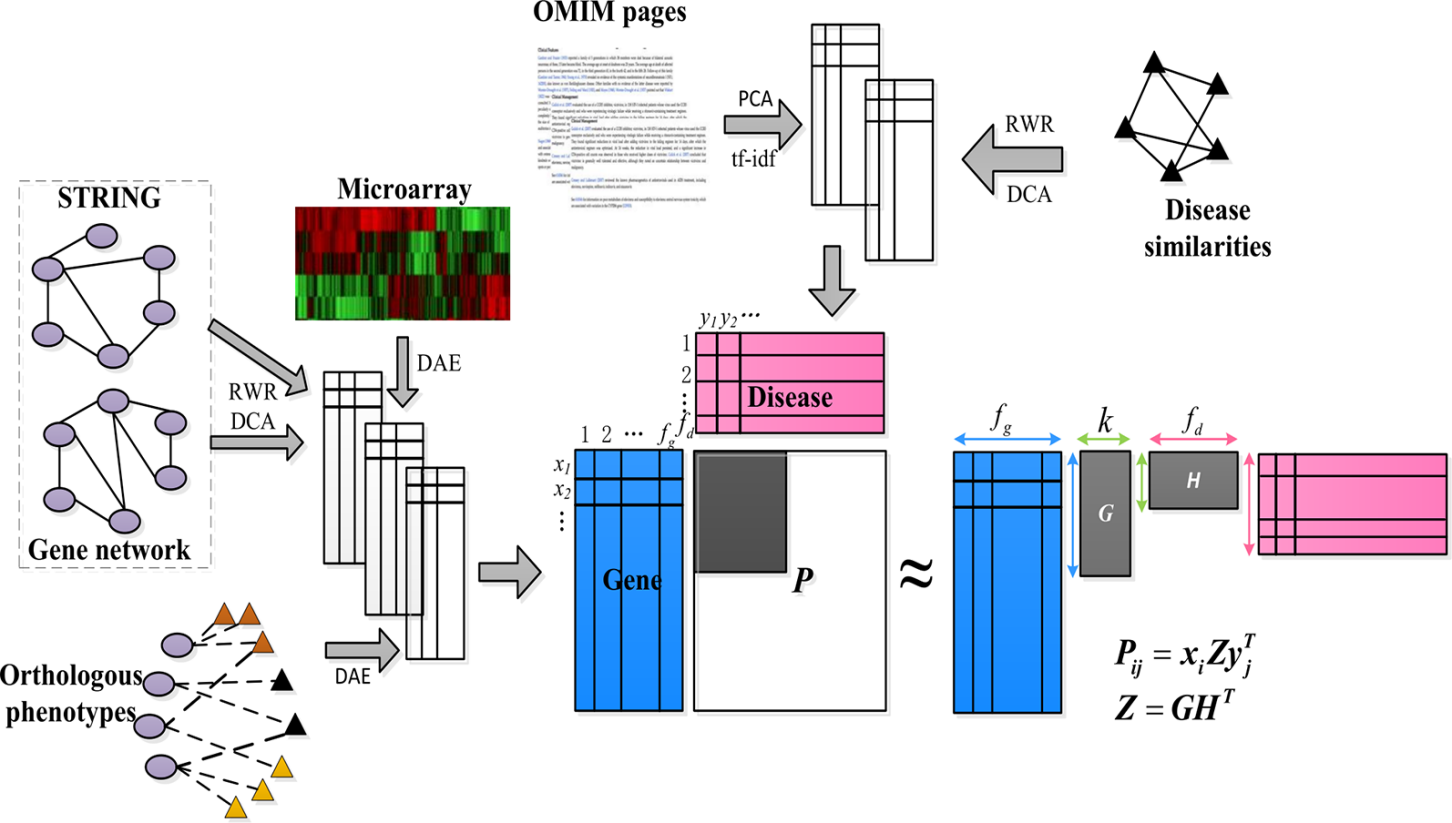
Schematic diagram of PUIMCHIF model framework.

We are interest in kinds of associations between the genes and diseases, but only part of them are known. So we want to make a prediction about the unknown pare from the known ones. As shown in **Figure 1**, our goal was to predict these unknown associations based on the constructed low-dimensional characteristics of the genes and diseases, and some known items in the gene–disease association matrix *P*, that is, to predict candidate genes potentially involved in the pathogenicity of the disease.

First, we constructed a low-dimensional eigenvector of genes and diseases from different biological sources (compact feature learning). We proposed different methods for learning compact features based on different forms of data. For the network data of genes and diseases, the random walk with restart algorithm (RWR) was first used to extract the diffusion state of each node in the network, and then DCA was used for dimensionality reduction to obtain the similarity of each gene (or disease) node in the heterogeneous network encoded by low-dimensional feature vectors. This is because genes (or diseases) with similar topological properties in the network are more likely to be functionally related.

Second, for common feature matrix data, to reduce the influence of high noise and data loss of biological data, we used denoising autoencoder (DAE) to reduce the dimensions of features.

Next, we applied the partial inductive matrix completion algorithm to predict the relationship between genes and diseases by combining the characteristics of multiple diseases and genes. One of the main advantages of this method is that it is generalized and can be applied to diseases that are not present during training, which cannot be predicted by traditional matrix completion methods. This allows us to take advantage of previous knowledge of known gene–disease interactions to predict unknown gene–disease interactions. Because we added an unbiased learning scheme for the unknown association relationship as a negative example, we finally adopted the PUIMC method for disease-causing gene prediction. The details of the PUIMCHIF algorithm are described below.

### Compact Feature Learning

In machine learning, the data are more important than the algorithm because the generalization of machine learning algorithm is about the ability from known data to the unknown data. Therefore, when we choose the prediction method based on machine learning to predict the disease-causing gene. First, we need to use high-quality data. Second, we need to conduct feature processing on the data to obtain more favorable data features for the prediction task.

We integrated a variety of biological data to extract characteristics of genes or diseases. Moreover, our goal was to obtain a low-dimensional effective data feature matrix, where one row of the feature matrix refers to a gene or disease, and the columns of the matrix represent different characteristics. The different compact feature learning methods that we used are described below.

#### RWR

Closely linked or functionally similar genes are more likely to cause the same or similar diseases. Random walk provides an effective framework for exploring relationships in networks.

Random walk with restart is referred to as RWR, which is a network diffusion algorithm widely used in the analysis of complex biological network data (Navlakha and Kingsford, 2010; Cao et al., 2014). Different from the traditional random walk method, each iteration of RWR introduces a predefined restart probability at the initial node, which can consider both local and global topological connection patterns within the network and take full advantage of direct or indirect relationships between nodes.

Here, matrix *A* and *B* are defined. Matrix *A* represents the weighted adjacency matrix of the interaction network of genes (or diseases). And in matrix *B* as shown in equation (1), each element *B*_*ij*_ describes the probability of transition from node *i* to node *j*. $s_{i}^{t}$ represents an *n*-dimensional distribution vector, and each element stores the probability that a node is accessed after iterating *t* times from node *i* during the random walk. The formula for calculating RWR is shown in equation (2).

(1) $$B_{ij}=\frac{A_{ij}}{{\text{Σ}}_{j^{\prime }}A_{ij^{\prime }}}$$

(2) $$s_{i}^{t+1}=\left(1-p_{r}\right)s_{i}^{t}B+p_{r}\delta_{i}$$

In equation (2), *δ*_*i*_ represents an *n*-dimensional standard basis vector and *δ*_*i*_(*i*) = 1, *δ*_*i*_ (*j*) = 0, for ∀*j* ≠ *i*. And *p*_*r*_ is a predefined restart probability that controls the relative influence of local structure and global structure in the diffusion process. With a higher value, more attention is paid to the local structure in the network.

For a node in the iterative process, we can obtain a stable distribution $s_{i}^{\infty }$, so we define *s*_*i*_ as the “diffusion state” of node *i*, that is $s_{i}=s_{i}^{\infty }$. The *j*th element *s*_*ij*_ of *s*_*i*_ represents the probability that the RWR starts from node *i* and ends at node *j* in equilibrium. When two nodes have similar diffusion states, it generally means that they are more similar than other nodes in the network and may have similar functions. This discovery provides a basis for predicting unknown gene–disease associations.

#### Diffusion Component Analysis

Although the diffuse states generated by the above RWR process represent the underlying topological environment and intrinsic connectivity spectrum of each gene or disease node in the network, they may not be completely accurate due to the low-quality and high-dimensional nature of biological data. For example, a small number of missing or false interactions in the network can significantly affect the outcome of the diffusion process (Kim and Leskovec, 2011). It is often inconvenient to directly use high-dimensional diffusion states as topological features in prediction tasks.

To solve this problem, we used a dimensionality reduction method called DCA to reduce the dimensions of the feature space and obtain important topological features from the diffusion state. In addition, for multi-omics networks, DCA also performs very well. The key idea of DCA is to obtain an informative but low-dimensional vector representation. Similar to principal component analysis (PCA), which seeks the inherent low-dimensional linear structure of data to best interpret variances, DCA learns the low-dimensional vector representation of all nodes to best interpret their patterns of connection in heterogeneous networks. We will briefly describe the DCA framework below.

To achieve the purposes of noise reduction and dimensionality reduction, DCA uses the polynomial logic model represented by a low-dimensional vector to approximate the obtained diffusion state distribution, and it has far fewer dimensions than the original *n*-dimensional vector representing the diffusion state. Specifically, the probability of assigning node *i* to node *j* in the diffusion state is modeled as:

(3) $${\hat{s}}_{ij}=\frac{\exp\left\{x_{i}^{T}w_{j}\right\}}{\sum_{j^{\prime }}\exp\left\{x_{i}^{T}w_{j^{\prime }}\right\}}$$

In equation (3), *x*_*i*_, *w*_*i*_∈*ℝ*^*d*^, *d* ≪ *n*. We take *w*_*i*_ as the context feature and *x*_*i*_ as the node feature of node *i*, both of which describe the topological properties of the network. If *x*_*i*_ and *w*_*i*_ point in similar directions, we obtain a larger inner product. This means that node *j* may be frequently visited in a random walk starting from node *i*. DCA uses the obtained diffusion state *S*={*s*_1_,⋯, *s*_*n*_ } as input to optimize *w* and *x* of all nodes. The optimization method uses KL divergence, as shown in equation (4).

(4) $$\underset{w,\;x}{\min}C\left(s,\;\hat{s}\right)=\underset{w,x}{\min}\frac{1}{n}\sum_{i=1}^{n}D_{KL}(s_{i}||{\hat{s}}_{i})$$

*D*_*KL*_(⋅||⋅) is the KL divergence between the two distributions. We use *w* and *x* to represent this formula according to the definition of KL invergence and $\hat{s}$.

(5) $$C\left(s,\hat{s}\right)=\frac{1}{n}\sum_{i=1}^{n}\left[H\left(s_{i}\right)-\sum_{j=1}^{n}s_{ij}\left(w_{i}^{T}x_{j}-\log\left(\sum_{j^{\prime }=1}^{n}\exp\left\{w_{i}^{T},x_{j^{\prime }}\right\}\right)\right)\right]$$

In equation (5), *H*(⋅) represents entropy. The objective function can find the low-dimensional vector representation of *w* and *x* using the standard quasi-Newton L-BFGS method. Although the obtained low-dimensional vector can effectively capture the network structure, we found that this optimization method is time-consuming.

To make the DCA framework more suitable for large biological networks, we use a more efficient method, clusDCA (Wang et al., 2015), which is based on matrix factorization, to decompose the diffusion states and obtain their low-dimensional vector representations. According to the definition, the following formula can be obtained:

(6) $$\begin{array}{c} \log{\hat{s}}_{ij}=x_{i}^{T}w_{j}-\log\sum_{j^{\prime }}\exp\left\{x_{i}^{T}w_{j^{\prime }}\right\} \end{array}$$

The first term corresponds to the low-dimensional approximation of ${\hat{s}}_{ij}$. The second term is a normalization factor, ensuring that ${\hat{s}}_{i}$ is a well-defined distribution. By removing the second term, we relax the constraint that the elements in ${\hat{s}}_{ij}$ must add up to 1. Although the obtained low-dimensional approximation of the diffusion state is no longer a strictly valid probability distribution, it is found that these approximations are very close to the true distribution, and the effects of relaxation are negligible. Therefore, it can be simplified as:

(7) $$\log{\hat{s}}_{ij}=x_{i}^{T}w_{j}.$$

In addition, we use the sum of squared errors as the objective function, instead of minimizing the relative entropy between the original diffusion state and the approximate diffusion state.

(8) $$\underset{w,\;x}{\min}C\left(s,\;\hat{s}\right)=\underset{w,x}{\min}\sum_{i=1}^{n}\sum_{j=1}^{n}{\left(w_{i}^{T}x_{j}-\text{log }s_{ij}\right)}^{2}$$

The obtained objective function can be optimized by singular value decomposition (SVD). To avoid taking the logarithm of 0, we add a small positive number $\frac{1}{n}$ to *s*_*ij*_. The calculation formula of the logarithm diffusion state matrix *L* is as follows:

(9) L=log(S+Q)−log(Q).

In equation (9), *S*∈*ℝ*^*n*×*n*^, *Q*∈*ℝ*^*n*×*n*^ and $Q_{ij}=\frac{1}{n}$, for ∀*i*, *j*. Using the singular value decomposition method, we decompose *L* into three matrices:

(10) $$L=U\Sigma V^{T}$$

In equation (10), *U*∈*ℝ*^*n*×*n*^, *V*∈*ℝ*^*n*×*n*^, Σ∈*ℝ*^*n*×*n*^ and Σ is a diagonal singular value matrix. To obtain the low-dimensional vectors *w*_*j*_ and *x*_*i*_ in d dimensions, we simply select the first d singular vectors *U*_*d*_, *V*_*d*_, and Σ_*d*_. Each row of matrix $X={\left[x_{1},\;\ldots ,\;x_{n}\right]}^{T}$ represents the low-dimensional eigenvector corresponding to each node in the network. In matrix $W={\left[w_{1},\;\ldots ,\;w_{n}\right]}^{T}$, each row represents the corresponding vector of the context feature. The formulas for calculating *X* and *W* are as follows:

(11) $$X=U_{d}{\text{Σ}}_{d}^{1/2},\;\;W=V_{d}{\text{Σ}}_{d}^{1/2}.$$

#### Denoising Autoencoder

Autoencoder is an unsupervised neural network model. It learns the implicit features of input data, which is called “coding.” At the same time, the original input data can be reconstructed with the learned new features, which is called “decoding.” Intuitively, autoencoder can be used for reducing feature dimensionality, like principal components analysis (PCA), but with stronger performance than PCA because the neural network model can extract more effective new features.

**Figure 2:**
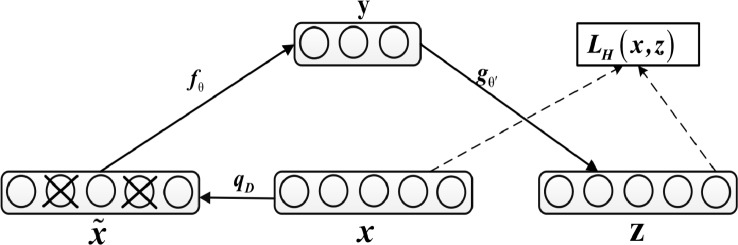
Diagram of Denoising Autoencoder.

The denoising autoencoder adds noise to the input *x* to obtain $\tilde{x}$, and after training, it obtains a noiseless output *z*, as shown in **Figure 2**.

This prevents the autoencoder from simply copying the input to the output, so as to extract useful patterns in the data and improve the weight robustness. Noise can be either pure gaussian noise added to the input or randomly discarding a feature at input layer, similar to dropout. The specific equation for calculating *z* is as follows:

(12) $$\begin{array}{c} y=f\left(\tilde{x}W_{1}+b_{1}\right)\\ z=g\left(yW_{2}+b_{2}\right) \end{array}$$

In addition, network parameters are trained to minimize reconstruction errors, namely:

(13) $$\min L_{H}\left(x,z\right)=\min||x-z_{p}.$$

### Pathogenic Gene Prediction Method

#### Standard Inductive Matrix Completion

In the gene–disease association matrix $P\in {\mathbb{R}}^{N_{g}\times N_{d}}$, each row represents a gene ID and the number of genes is *N*_*g*_. Each column represents a disease phenotype and the number of diseases is *N*_*d*_. If *P*_*ij*_ = 1, this means that gene *i* is related to disease *j*, and *P*_*ij*_ = 0 means that the relationship between gene *i* and disease *j* is uncertain. Based on the most successful and deeply studied matrix completion method in the recommender systems, the IMC algorithm was used to complete the task of learning gene–disease associations. The advantage of this is that this method is inductive, and it can achieve the prediction of new genes or diseases that have rarely been studied.

**Figure 3:**
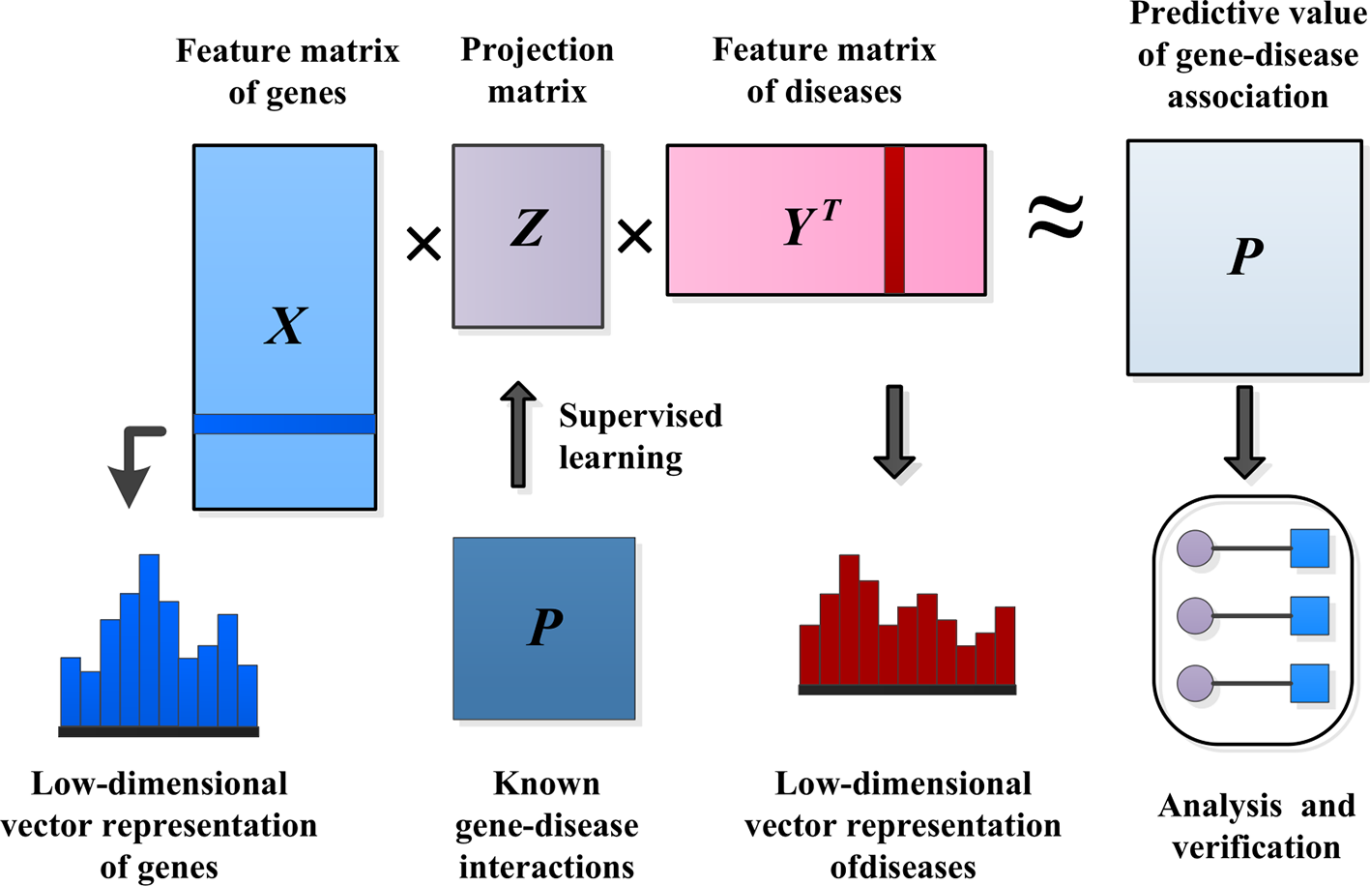
Methods of predicting pathogenic genes.

IMC assumes that the association matrix has a low rank, with the goal of recovering *Z* using the observed values of *P* and the eigenvectors of genetic diseases, as shown in **Figure 3**.

The eigenvector matrix of *N*_*g*_ genes is represented by $X\in {\mathbb{R}}^{N_{g}\times f_{g}}$, and the eigenvector of gene *i* is represented by $x_{i}\in {\mathbb{R}}^{f_{g}}$. Similarly, $Y\in {\mathbb{R}}^{N_{d}\times f_{d}}$ is used to represent the eigenvector matrix of *N*_*d*_ diseases, and $y_{i}\in {\mathbb{R}}^{f_{d}}$ is used to represent the eigenvector of disease *j*. The inductive matrix completion problem is to recover a low-rank matrix *Z* by using the known association Ω^+^ from the gene–disease association matrix *P*. We established a bilinear function to learn the projection matrix *Z* between the gene space and the disease space to predict the interaction between unknown genes and diseases. We modeled the matrix *P* as *XZY*^*T*^≈ *P*. Then, we used the following formula to measure the probability of pairwise interaction score between gene *i* and disease *j*, and the higher the score(*i*, *j*) value, the more likely gene *i* and disease *j* interact.

(14) $$score\left(i,j\right)=x_{i}Zy_{j}^{T}$$

There is usually a significant correlation between spatially close eigenvectors of genes or diseases, which can greatly reduce the number of effective parameters needed to model gene–disease interactions in *Z*. To consider this problem, we applied a low-rank constraint on *Z* and learned only a few potential factors. Let *Z* = *GH*^*T*^, where $G\in {\mathbb{R}}^{f_{g}\times k}$, $H\in {\mathbb{R}}^{f_{d}\times k}$, and *k* is small. This low-rank constraint not only alleviates the overfitting problem, but also facilitates the process of optimizing the calculation (Wang et al., 2015). The optimization problem of low-rank constraint is NP-hard on the original matrix *Z*. One standard method of relaxing the low-rank constraint is to minimize the trace norm, that is, the sum of the singular values. Minimizing the trace norm of *Z* = *GH*^*T*^ is equivalent to minimizing $\frac{1}{2}\left({\left\|G\right\|}_{F}^{2}+{\left\|\right\|}_{F}^{2}\right)$. The decomposition of *Z* into *G* and *H* solves the following optimization problems by alternating minimization. A common choice for the loss function *ℓ* is the square loss function. *λ* is the regularization parameter.

(15) $$\min\sum_{\left(i,j\right)\in {\text{Ω}}^{+}}\ell \left(P_{ij},x_{i}^{T}GH^{T}y_{i}\right)+\frac{\lambda }{2}\left({\left\|G\right\|}_{F}^{2}+{\left\|H\right\|}_{F}^{2}\right)$$

#### Improved Inductive Matrix Completion

To optimize the objective function, we introduce the idea of PU-Learning. Although we predicted positive examples from unknown relationships, that is, candidate disease-causing genes, it was undeniable that these unknown genes-disease pairs may be unrelated. Therefore, unknown association relationship information was added to the learning process as a negative example, and the objective function was as follows:

(16) $$\min\sum_{\left(i,j\right)\in {\text{Ω}}^{+}}\ell \left(P_{ij},x_{i}^{T}GH^{T}y_{i}\right)+\alpha \sum_{\left(i,j\right)\in {\text{Ω}}^{-}}\ell \left(P_{ij},x_{i}^{T}GH^{T}y_{i}\right)+\frac{\lambda }{2}\left({\left\|G\right\|}_{F}^{2}+{\left\|H\right\|}_{F}^{2}\right)$$

We represent the unknown association in the gene–disease association matrix *P* as Ω^−^. The key parameter α < 1 because the penalty weight of the known relationship must be greater than the unknown relationship. Finally, equation (14) was still used to calculate the interaction score between gene *i* and disease *j*. The scores are sorted in descending order, and the first *k* genes were selected as candidate pathogenic genes for the corresponding disease.

## Data Sets and Features

The data sets used in this paper can be divided into three categories: gene–disease association data, gene characteristic data, and disease characteristic data.

### Gene–Disease Associations

The known gene–disease association data that we used were from the OMIM database, which contained 12,331 genes, 3,209 diseases, and 3,954 known gene–disease associations (the total number of nonzero elements in the gene–disease association matrix). It can be seen that the data in the incidence matrix are very sparse, with more than 90% of the columns having only one nonzero item and 70% of the rows having no nonzero elements.

### Gene Characteristics

Gene characteristics were obtained by processing four different data sources through compact feature learning (*Compact Feature Learning*). The first source of gene characteristics was gene microarray data, which contained 8,755 genes and 4,536 characteristics. First, we linearly transformed the expression range of each gene to between 0 and 1. Because these characteristics are highly correlated, we used four layers of denoising autoencoder to reduce the dimensionality of the data, and the number of cells in each hidden layer was 3,000-800-300-100, respectively. Moreover, gaussian noise with a noise factor of 0.2 was added to the input data, and sigmoid was used to activate each layer. The model was optimized with Adam, and epoch was 100.

The second source of gene characteristics was from homologous gene phenotypic associations in eight other species, which were more abundant than in studies of human genetic diseases. The data used in the experiment are shown in **Table 1**. The features were extracted by two-layer denoising autoencoder with the following specific parameters: the number of nodes in each layer is “200–100,” the corruption level of data is 0.2, the activation function is sigmoid function, the batch size is set as 150, and the model is optimized by Adam.

**Table 1:** Species Details.

Number	Species name	Number of disease phenotypes	Number of associations
1	Human	3209	3954
2	Arabidopsis thaliana	1137	12010
3	Worm	744	30519
4	Drosophila	2503	68525
5	Zebrafish	1143	4500
6	Escherichiacoli	324	72846
7	Gallus	1188	22150
8	Mouse	4662	75199
9	Saccharomyce	1243	73284

In addition, the data on interactions between genes can also be used as a part of the characteristics of genes. We integrated two networks, HumanNet (Lee et al., 2011) and STRING (Szklarczyk et al., 2017), for unified analysis. These two sets of data represent gene–gene interaction networks, but there are differences between them (Kuang et al., 2018). The integrated analysis of different sets of data can verify each set, and they can help to validate each other and expand understanding the potential rules. We used the RWR and DCA methods to fuse two networks to extract gene features. We set the restart probability to 0.05 and extracted the 600-dimensional gene characteristics. Finally, the gene characteristics used in the model were 800 dimensions.

### Disease Characteristics

The disease characteristics are mainly derived from two data sources: the disease similarity network MimMiner and clinical manifestation data of the disease, as well as a large amount of data from analysis of the medical literature.

MimMiner data are processed by literature (van Driel et al., 2006) and are freely available online. This data set has been applied in gene prioritization methods (Vanunu et al., 2010; Singh-Blom et al., 2013; Natarajan and Dhillon, 2014). RWR and DCA were used to extract 100-dimension disease features in the disease similarity network, and the restart probability was set as 0.05.

Another disease feature that we incorporated was from the OMIM disease webpage. We paid special attention to the clinical features and clinical management of webpages. We obtained disease features through text mining. We used PCA to reduce the dimensions of feature space and retained the first 100 principal components. Finally, we obtained 200-dimension disease characteristics.

## Experiment

*Evaluation Indexes and Methods* introduces the evaluation indexes and methods of the experiment. *Parameter Settings* describes the influence of important parameters in the experiment. In *Global Performance*, the global performance of the experiment is compared. *Prediction of New Genes and New Diseases* compares the ability to predict new genes and new diseases. *Newly Discovered Genes* compares the ability to predict newly discovered associations.

### Evaluation Indexes and Methods

In the experiment, to quantitatively evaluate our method and compare it with the most advanced disease-causing gene prioritization methods, we used a cross-validation strategy to measure gene recovery. We divided the known gene–disease pairs into three groups of the same size. The associations in one group were hidden, and the associations in the remaining two groups were used as training data, repeated three times to ensure that each group was hidden only once. For each disease in our data set, we ranked all of the genes according to the degree to which they were associated with the disease. The first *r* genes were taken as candidate pathogenic genes for corresponding diseases; namely, the top-*r* ranking method was used. The performance of the algorithm was analyzed by comparing the recall and precision of each method under different thresholds *r*, usually *r* ≤ 100. The formula for calculating this was as follows:

(17) $$\begin{array}{c} Recall=\frac{\text{TP}}{\text{TP}+\text{FN}} \end{array}$$

(18) $$\begin{array}{c} Precision=\frac{\text{TP}}{\text{TP}+\text{FP}} \end{array}$$

Recall rate refers to the proportion of positive cases correctly judged by the model relative to all positive cases (TP+FN) in the data set. FN represents the data that are mistaken as negative cases by the model but are actually positive cases. The precision rate is the proportion of true positive cases (TP) relative to all positive cases (TP+FP) judged by the model (Xiong et al., 2012; Xu et al., 2017; Cheng et al., 2019; Cheng et al., 2019).

To further confirm the value of our approach, we also used the mean percentile ranking (MPR), an evaluation index based on recall, to evaluate the performance of the algorithm. This evaluation index has been applied in recommendation algorithm and analyses of the performance for predicting drug-targets (Hu et al., 2008; Johnson, 2014; Li et al., 2015; Ding et al., 2017; Hao et al., 2019; Liu et al., 2019b; Liu et al., 2019c; Zeng et al., 2019) and disease biomarkers (Chen et al., 2016; Zeng et al., 2016; Hong et al., 2019; Xu et al., 2019). For each disease, the genes were ranked in descending order according to the calculated gene–disease predictive value. The average ranking of the true and established associations among them is the final result. Here, rank_*ji*_ can be used to represent the percentile ranking (PR) of gene *j* and disease *i*. rank _*ji*_ = 0*%* indicates that disease *i* is most likely to interact with gene *j*. Similarly, rank _*ji*_ = 100*%* indicates that disease *i* has the lowest probability of interacting with gene *j*. Therefore, the definition of MPR is as follows:

(19) $$\begin{array}{c} MPR=\frac{\sum_{i=1}^{N_{D}^{t}}R_{i}}{N_{D}^{t}} \end{array}$$

$N_{T}^{t}$ represents the number of diseases in the test set, and the formula for calculating *R_i_* is as follows:

(20) $$\begin{array}{c} R_{i}=\frac{\sum_{j=1}^{N_{T}^{t}}{\text{rank}}_{ji}}{N_{T}^{t}} \end{array}$$

$N_{T}^{t}$ represents the number of genes in the test set for current disease *i*. It is important to emphasize that lower MPR values are preferable because they indicate that our approach has a higher probability, which means that the model works better. Conversely, a higher MPR indicates a lower likelihood of gene interactions with disease. Clearly, the randomly generated list is expected to have an MPR of 50%. Using this measure, we can obtain a list of recommended candidate pathogenic genes, where the recommended optimal prediction is used for higher priority experimental validation.

### Parameter Settings

**Figure 4:**
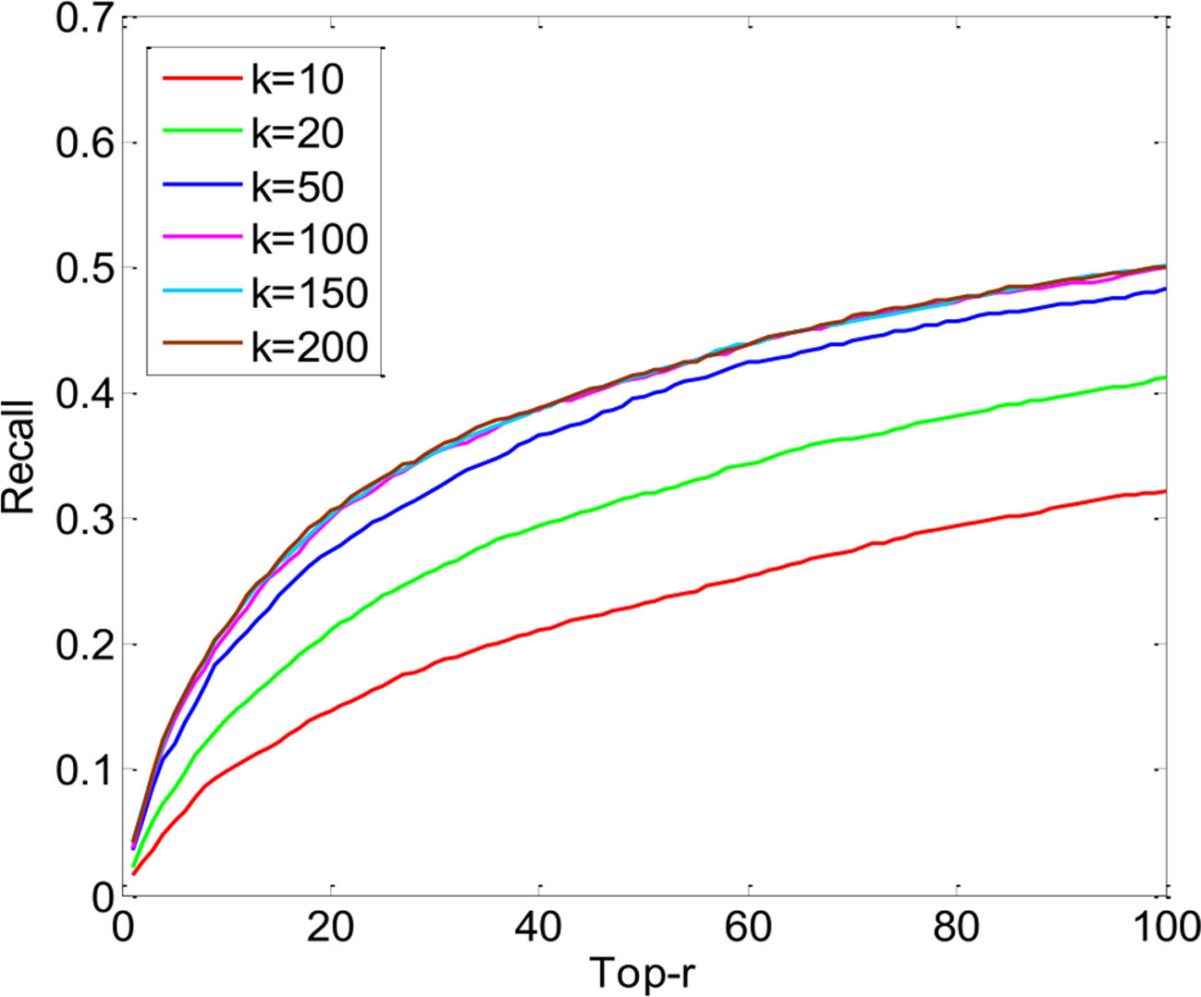
Performance Comparison of PUIMCHIF with Different *k* Values.

The key parameters of PUIMCHIF are the rank *k* of matrix *Z*∈*ℝ*^800×200^, the regularized parameter *λ*, and the penalty weight *α* for the unknown relation. As can be seen from **Figure 4**, the performance of the PUIMCHIF method increases with the increase of *k*. When *k* = 100, 150, and 200, the three curves are very close. In the following experiment, the PUIMCHIF method uniformly set parameters as follows: *k* = 200, *λ* = 0.02, and *α*=0.0035.

**Figure 5:**
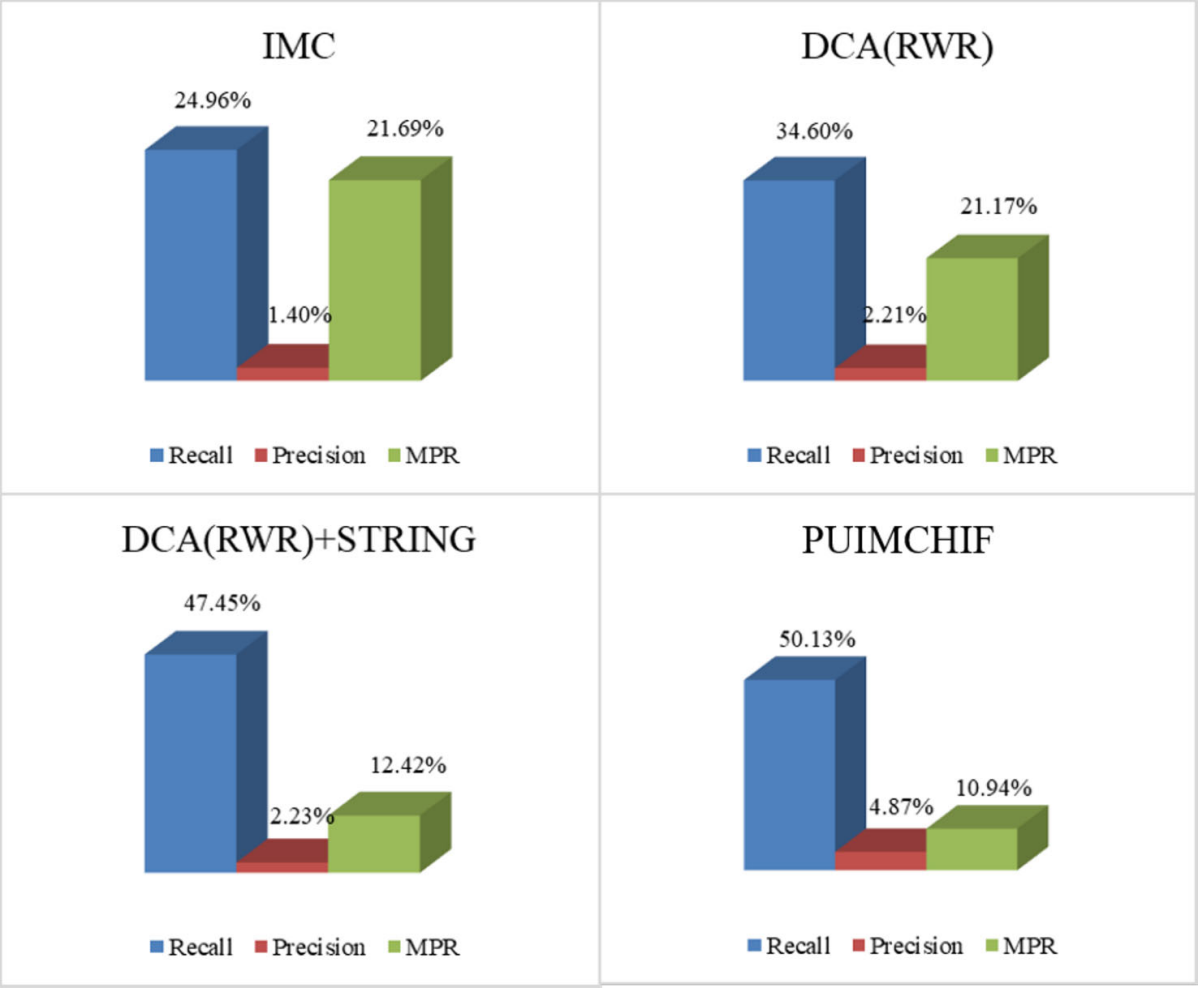
Model Optimization Results.

As mentioned earlier, our approach features four improvements over the original IMC approach. For **Figure 5**, recall, precision, and MPR were used to analyze the effect of our improved method. The four experimental results in the figure represent (a) the initial experimental results of the original IMC method, (b) the results of extracting features by using RWR and DCA, instead of PCA, for the network data of diseases and genes, (c) the prediction results of adding STRING data to the gene interaction network, and (d) the experimental results of each index of the PUIMCHIF method.

We found that using RWR and DCA can better extract the gene–gene and disease–disease relationships, and helps to improve the prediction of candidate pathogenic genes. Meanwhile, it was also found that the protein interaction network STRING improved the prediction recall rate to 47.45%, and the MPR value also decreased significantly. Using denoising autoencoder to represent the characteristics of genes and diseases, and introducing the idea of PU-Learning into the inductive matrix completion can further improve the predictive performance.

### Global Performance

**Figure 6:**
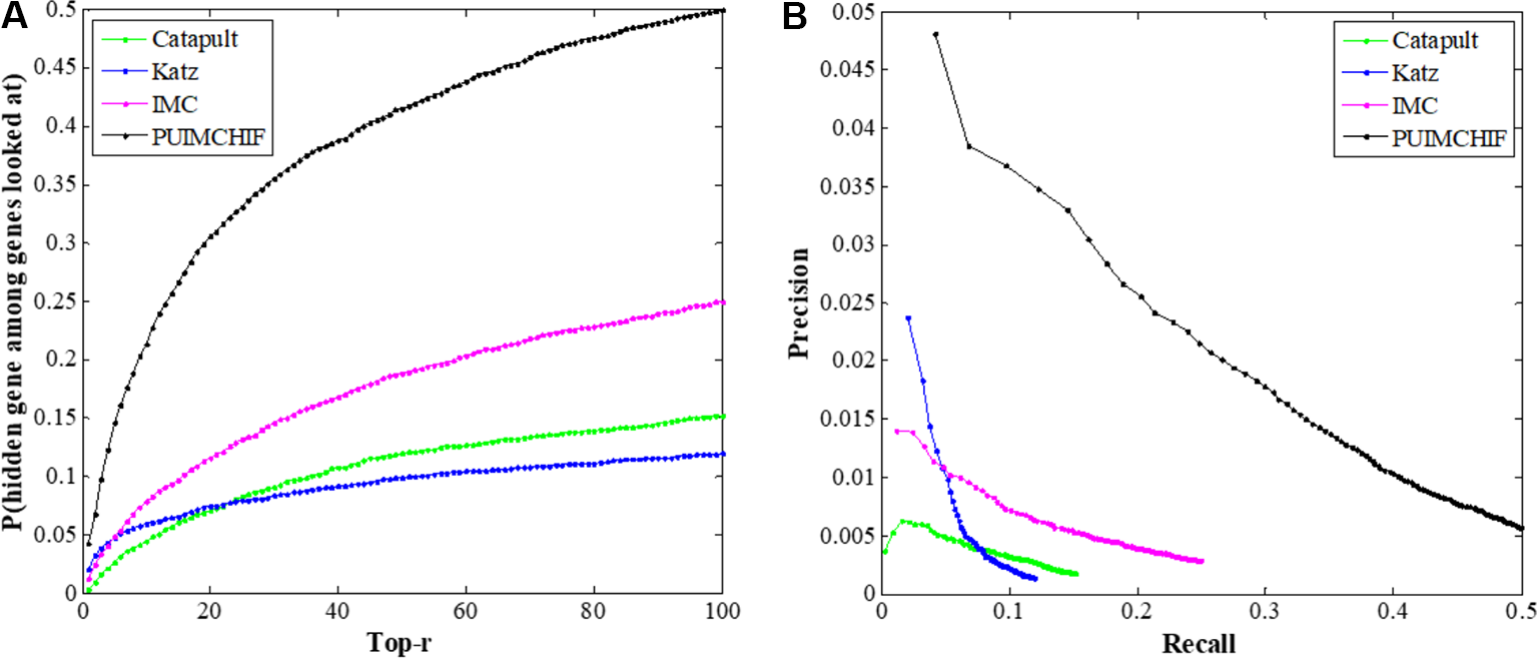
Global Performance with Different Thresholds *r*. **(A)** Recall rate at different threshold *r*. **(B)** Precision-recall curve.

In this experiment, the threefold cross-validation method was used to compare the overall performance of the proposed method with CATAPULT, Katz, and IMC. As shown in **Figure 6A**, the vertical axis gives the probability of recovering the true gene association in the top-r prediction of different r values on the horizontal axis. The experimental results show that the PUIMCHIF algorithm proposed in this paper has a much higher probability of recovering true gene associations under different thresholds than the other methods. **Figure 6B** presents the precision–recall curve.

In addition, **Table 2** shows the results of three evaluation indexes for each method when the threshold *r*=100. It is worth mentioning that a smaller value of MPR is associated with a higher probability and a better effect. It can be seen that the MPR value of PUIMCHIF is the lowest and the recall rate reaches 50%, while the best method among other methods, IMC, is only 25%, that is, the recall is doubled. The precision rate was also twice that of Katz which is the best method of other methods, reaching 4.87%. The overall performance of PUIMCHIF has been further improved, confirming the superiority of our method.

**Table 2:** Experimental Results with Threshold *r* =100.

Methods	Recall	Precision	MPR
CATAPULT	0.152251	0.006289	0.319410
Katz	0.120132	0.023752	0.335564
IMC	0.249621	0.014036	0.216856
PUIMCHIF	0.501265	0.048681	0.109412

### Prediction of New Genes and New Diseases

#### Prediction of New Genes

**Figure 7:**
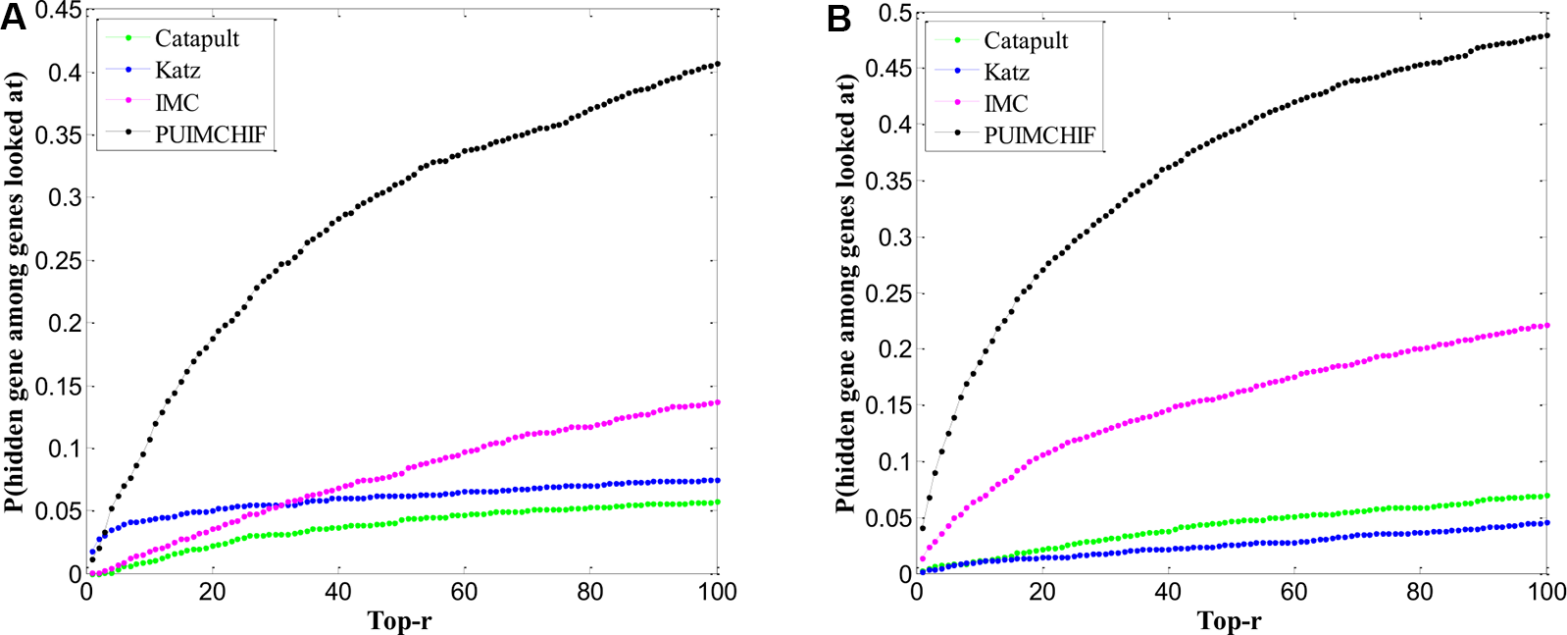
Prediction of New Genes and New Diseases. **(A)** Prediction of New Genes. **(B)** Prediction of New Diseases.

One problem affecting prioritization assessments is that well-related genes and diseases tend to be more predictable and therefore tend to generate inflated recall rates. Here, we focued only on genes that are known to have a single association in the gene-disease association data set. In other words, we selected the gene corresponding to the row with only 1 non-zero element in the gene-disease association matrix as the validation set, and hided these known associations in the training process. After repeated three-fold cross validations, **Figure 7A** shows the predictive power of different methods within the threshold *r* < 100. The Y-axis represents the probability of a true known single gene association hidden during recovery training.

**Table 3** shows the specific experimental results of each method when *r* = 100. For the prediction of new genes, although the precision rate was slightly lower than Katz, the recall rate of our PUIMCHIF was significantly higher than other methods, reaching 40.7% when the recall rate of IMC method was only 13.7%. At the same time, we found that using the MPR index to evaluate the results, the PUIMCHIF method was only 13.5%, much lower than Katz and CATAPULT. This also shows that our method is more reliable.

**Table 3:** Prediction of New Genes with Threshold *r* =100.

Methods	Recall	Precision	MPR
CATAPULT	0.056943	0.001227	0.497410
Katz	0.074838	0.018446	0.466105
IMC	0.137195	0.001935	0.284610
PUIMCHIF	0.407281	0.013840	0.135043

#### Prediction of New Diseases

Similar to the prediction of new genes, we only considered diseases with a single known association in the gene-disease association data set as the validation set, that is, diseases corresponding to the columns with only 1 non-zero element in the gene-disease association matrix, and hided these known associations during training. Similarly, a three-fold cross-validation analysis was used, and the results are shown in **Figure 7B**. The probability that the proposed method could recover the true association of new diseases reached 48%, which was a significant improvement compared with other methods. Moreover, the MPR value of our method was lower than that of other methods, and the precision rate was nearly 2.7 percentage points higher than that of IMC method. As can be seen from **Table 4**, PUIMCHIF method is superior to other methods in three evaluation indexes.

**Table 4:** Prediction of New Disease with Threshold *r* =100.

Methods	Recall	Precision	MPR
CATAPULT	0.070060	0.002392	0.346974
Katz	0.045454	0.001709	0.363452
IMC	0.221804	0.014012	0.226905
PUIMCHIF	0.479836	0.040671	0.112801

### Newly Discovered Genes

Cross-validation of retrospective data can lead to overly optimistic performance estimates. For example, certain gene interactions may be found because of associations with specific diseases being evaluated. Although the association itself is hidden, other features are contaminated by this information. Therefore, the use of recently reported associations to assess gene prioritization tools is unbiased in this assessment.

**Figure 8:**
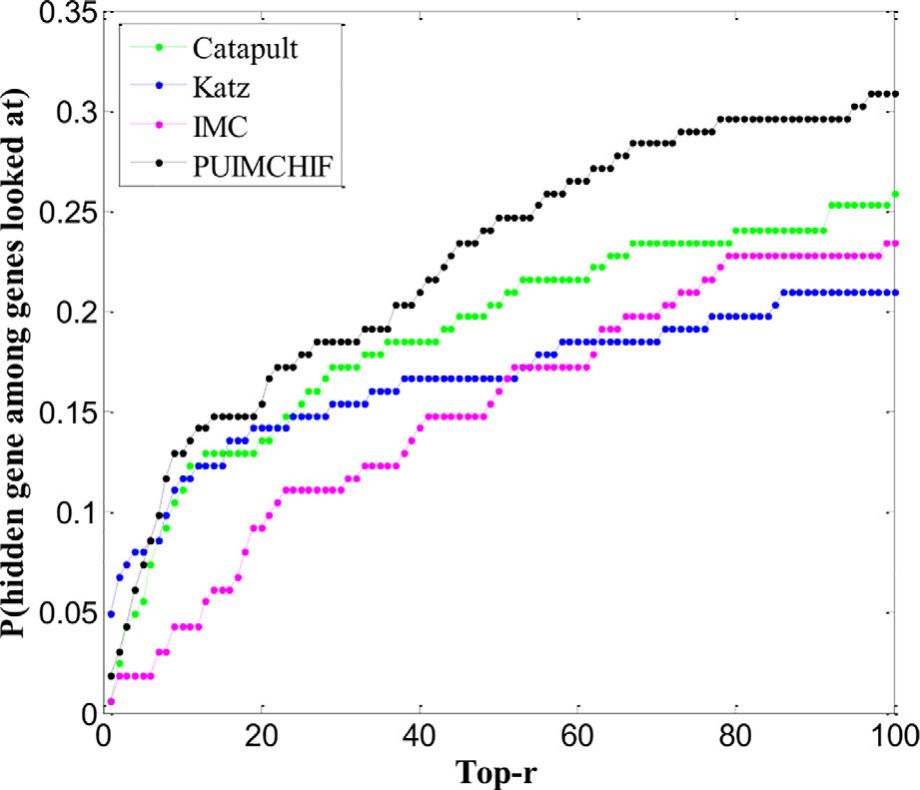
Newly Discovered Prediction of Association.

We trained all methods using all the gene associations of the 3,209 OMIM diseases collected. We found 162 newly discovered associations, of which 83 genes had no known associations previously. Thus, the assessment of new associations also helps determine the ability of methods to recommend new genes. The ranking performance of each method in 162 new associations is shown in **Figure 8**. We can see that the IMC method is superior to other methods in the range of threshold 6 ≤ *r* ≤ 100.

## Conclusion

In this paper, a PU induction matrix completion algorithm based on heterogeneous information fusion, PUIMCHIF, was proposed to predict gene–disease associations. Based on the specific advantages of IMC method, PUIMCHIF can predict new genes and diseases, and has good predictive performance. In addition, because closely connected or functionally similar genes are more likely to cause the same or similar diseases, we constructed low-dimensional feature representations of genes and diseases from various data sources such as STRING using the compact feature learning method, which effectively alleviated the impact of data sparsity. Although there is no evidence that genes are unrelated to diseases in the data set, it is clear that most of the unknown associations are negative. PUIMCHIF conducts biased learning by treating unlabeled data as negative cases and constraining the penalty weight of known relationships to be greater than that of unknown relationships. Compared with the existing prediction methods, the PUIMCHIF method can significantly improve the prediction results regarding recall rate, precision rate, and MPR. According to the evaluation index of MPR, the experimental results of the PUIMCHIF method that we proposed are the lowest; that is to say, the candidate genes given by our algorithm have a higher priority for validation by biological experiments.

## Data Availability Statement

The datasets analyzed in this article are not publicly available. Requests to access the datasets should be directed to chunyu@hit.edu.cn.

## Author Contributions

CW initiated the idea, conceived the whole process and drafted the manuscript. JZ and XW implemented the experiments and designed the figures. KH helped with data analysis and revised the manuscript. MG and finalized the paper. All authors have read and approved the final manuscript.

## Conflict of Interest

The authors declare that the research was conducted in the absence of any commercial or financial relationships that could be construed as a potential conflict of interest.
